# Organoid Models of Glioblastoma and Their Role in Drug Discovery

**DOI:** 10.3389/fncel.2021.605255

**Published:** 2021-02-05

**Authors:** Matthew J. Rybin, Michael E. Ivan, Nagi G. Ayad, Zane Zeier

**Affiliations:** ^1^Department of Psychiatry and Behavioral Sciences, University of Miami Miller School of Medicine, Miami, FL, United States; ^2^Center for Therapeutic Innovation, University of Miami Miller School of Medicine, Miami, FL, United States; ^3^Sylvester Comprehensive Cancer Center, University of Miami Miller School of Medicine, Miami, FL, United States; ^4^Department of Neurological Surgery, University of Miami Miller School of Medicine, Miami, FL, United States

**Keywords:** glioblastoma, brain organoids, organoid-GBM modeling, drug discovery, compound screening

## Abstract

Glioblastoma (GBM) is a devastating adult brain cancer with high rates of recurrence and treatment resistance. Cellular heterogeneity and extensive invasion of surrounding brain tissues are characteristic features of GBM that contribute to its intractability. Current GBM model systems do not recapitulate some of the complex features of GBM and have not produced sufficiently-effective treatments. This has cast doubt on the effectiveness of current GBM models and drug discovery paradigms. In search of alternative pre-clinical GBM models, various 3D organoid-based GBM model systems have been developed using human cells. The scalability of these systems and potential to more accurately model characteristic features of GBM, provide promising new avenues for pre-clinical GBM research and drug discovery efforts. Here, we review the current suite of organoid-GBM models, their individual strengths and weaknesses, and discuss their future applications with an emphasis on compound screening.

## Hallmarks of GBM

Glioblastoma (GBM) is the most common and most aggressive brain cancer in adults. In 2014, the annual incidence of GBM was reported to be 3.19 cases per 100,000 people in the United States (Thakkar et al., 2014). However, incidence is rising due to aging of the population (Hoffman et al., 2006; Grech et al., 2020). The current standard of care is maximal surgical resection followed by radiation and chemotherapy with temozolomide (TMZ), a DNA alkylating agent. Notwithstanding these aggressive treatment measures, current median survival post-diagnosis is <2 years [Johnson and O'Neill, 2012; Witthayanuwat et al., 2018; for review of treatment strategies and drug repurposing for GBM see Carlsson et al. (2014) and Tan et al. (2018)].

A characteristic feature of GBM confering treatment resistance is vast inter- and intra-tumor heterogeneity (Hu et al., 2017; Skaga et al., 2019; Wenger et al., 2019). Recent discoveries have uncovered several determinants of GBM heterogeneity that include genetic abnormalities, altered epigenetic landscapes, transcriptional dysregulation, microenvironmental cues and developmental status (Patel et al., 2014; Neftel et al., 2019). Each of these features can vary dramatically between different patient tumors and even within different regions of the same tumor. Currently, a major goal of research efforts is to delineate the mechanistic determinants of GBM heterogeneity and develop model systems that faithfully recapitulate disease complexity. To this end, further classification of GBM subtypes and cellular states is needed. Using bulk sequencing, GBM tumors have been stratified into three major subtypes: proneural, classical, and mesenchymal (Verhaak et al., 2010; Wang et al., 2017). Using single-cell RNA sequencing (scRNA-seq), individual GBM cells can be stratified into four distinct cellular states: neural-progenitor-like (NPC-like), oligodendrocyte-precursor-like (OPC-like), astrocyte-like (AC-like), and mesenchymal-like (MES-like) (Neftel et al., 2019). However, these cellular states are plastic, thereby complicating tumor classification (Neftel et al., 2019).

Another hallmark of GBM is poorly defined tumor margins and prolific invasion into healthy brain tissues (Matsukado et al., 1961). While GBM rarely metastasizes outside of the brain, aggressive invasion within the brain parenchyma and perivascular space is typical. While the determinants GBM invasion remain largely unknown, emerging evidence supports two major mechanisms: protease-mediated invasion and hydrodynamic invasion. For protease-mediated invasion, GBM infiltration into the brain parenchyma is facilitated by extensive degradation of the extracellular matrix [for review of protease-mediated invasion in GBM see Mentlein et al. (2012)]. This mechanism is supported by multiple lines of evidence. For instance, GBM invasiveness is correlated with expression of ECM-degrading proteases, including matrix metalloproteases (MMPs) (Stojic et al., 2008). Moreover, protease inhibition attenuates invasion of established GBM cell lines using *in vitro* invasion assays (scratch wound healing and trans-well migration) (Chetty et al., 2012; Chou et al., 2015). The second and more recently proposed mechanism suggests that GBM infiltration is achieved by extreme shrinking and contorting of the cell driven by dynamic fluctuations in water through aquaporin channels in the plasma membrane. According to this theory, changes in ion gradients cause water to be extruded from cellular processes allowing for dramatic alterations of cell volume and increased cellular motility in extracellular spaces [for review see Cuddapah et al. (2014)]. Multiple observations support the hydrodynamic invasion model: (1) time-lapse imaging shows that invading GBM cells undergo dramatic morphology and volume changes (Watkins and Sontheimer, 2011), (2) invasiveness correlates with the expression of ion transporters that drive water exchange in GBM (Garzon-Muvdi et al., 2012), and (3) genetic or pharmacological inhibition of these ion channels reduces GBM infiltration (Soroceanu et al., 1999; Haas and Sontheimer, 2010; Lui et al., 2010; Garzon-Muvdi et al., 2012). Despite increasing knowledge of the molecular features of GBM invasion, drugs targeting GBM invasion have not yet succeeded in clinical trials [for review of early promising MMP-related clinical trials see Coussens et al. (2002)], although additional trials are ongoing (e.g., NCT04214392).

Both cellular heterogeneity and invasiveness contribute to the inability to manage GBM by complicating the development of effective pharmacotherapies and preventing complete surgical resection, thereby making recurrence inevitable. To overcome these challenges, new experimental models of GBM that more accurately recapitulate the true complexity of *in situ* GBM are badly needed. Here we provide an overview of the current GBM model systems to point out important limitations that have stymied therapeutic development. We then review the recent progress that has been made to develop novel human-specific organoid-GBM model systems that may help to overcome some of these limitations. Lastly, we highlight the current and future applications of organoid-GBM models with an emphasis on drug discovery.

## Established GBM Models

### *In vitro* GBM Models

One of the most commonly used glioblastoma cell lines, U87, was established over 50 years ago (Pontén and Macintyre, 1968). Similar lines have also been used extensively in recent decades as *in vitro* pre-clinical models of GBM. However, use of these established lines is increasingly controversial due to important differences when compared to *in situ* GBM (Gillet et al., 2011; Allen et al., 2016). A major milestone in the transition away from glioblastoma cell lines was the work of Lee et al. who compared primary GBM cells cultured under two different conditions: as a traditional cell line (with serum) or in conditions optimized for glioma stem cells (GSCs). They found that the genotype and gene expression profile of GSCs were more similar to patient tumors than cell lines established from the same original sample. Moreover, when injected into the brains of mice, GSCs were more tumorigenic and invasive than the cells cultured with serum. While the concept of cancer stem cells (CSCs) already existed at that time [Singh et al., 2003; see also review by Pardal et al. (2003)], the work of Lee and colleagues emphasized the importance of using GSCs to accurately model GBM and launched the era of primary 3D GSC spheroids. Now, GSCs are recognized to play important roles in GBM resistance to therapy (Bao et al., 2006; Chen et al., 2012). As such, GSCs (as 2D cultures and 3D spheroids) are commonly utilized in GBM research [Lathia et al., 2010; Suvà et al., 2014; Jin et al., 2016; Tirosh et al., 2016; Lan et al., 2017; Wang et al., 2018; Sachdeva et al., 2019; Trong et al., 2020; for review of recent GSC-related work consider Gimple et al. (2019), Prager et al. (2020), and Suvà and Tirosh (2020)]. While important discoveries have been made using these systems, GSC cultures (both 2D and 3D) have notable limitations. For example, GSCs cultured in 2D with serum for extended periods of time change morphology and lose their characteristic stem-like phenotype (Lee et al., 2006). Another limitation is that GSC cultures are relatively homogeneous and do not, therefore, recapitulate the complex cellular and microenvironmental interactions that are implicated in tumor initiation, invasion, resistance and recurrence (Calabrese et al., 2007; Lathia et al., 2010, 2012; Cheng et al., 2013; Zhou et al., 2015; Wang et al., 2018). To address the need for GBM model systems that more accurately reproduce these interactions, complex 3D organoid-GBM model systems have been developed.

### *In vivo* GBM Models

The need for human tissue-based 3D-GBM model systems is necessitated, in part, by the limitations of traditional *in vivo* systems. Current murine models of GBM include transgenic and patient-derived xenografts (PDX) [for review see da Hora et al. (2019) and Robertson et al. (2019)]. Transgenic models—typically genetically engineered mice—have been vital for understanding how oncogenic mutations contribute to tumor initiation and progression, especially in immune competent mice (Zhu et al., 2005; Marumoto et al., 2009). The foremost limitation of these transgenic models is the inability to recapitulate the complex genetic and phenotypic heterogeneity and cellular hierarchy observed in patient tumors. Meanwhile, PDX models are currently the gold standard for GBM modeling because they accurately recapitulate patient tumors including histological markers and invasiveness (Wang et al., 2009). PDX models typically utilize freshly resected tumors or GSCs cultured for varying amounts of time to introduce a fluorescent marker protein or other genetic modification. In either case, GBM cells can be xenografted orthotopically into the brain or passaged in the flank of immunocompromised mice. The benefits of orthotopic PDX models are that tumors grow in a 3D brain microenvironment (albeit non-human), are vascularized, and recapitulate key GBM phenotypes including invasion—even after multiple PDX passages in the flank of mice. The major drawbacks of PDX models are the time and cost of establishing and maintaining a PDX model and recent evidence suggests that extended PDX culture favors mouse-specific tumor evolution (Ben-David et al., 2017). Importantly, both transgenic and PDX murine models lack a human-specific brain microenvironment which plays an increasingly recognized role in GBM (Pine et al., 2020).

To address important limitations of previous *in vitro* and *in vivo* GBM models, several groups have developed sophisticated organoid-GBM culture systems. These emerging research tools are advantageous in that they more faithfully recapitulate GBM characteristics such as cell-type heterogeneity and microenvironmental factors (with the potential for patient-specific modeling). Improved disease relevance and scalability are key attributes that have generated great interest in their potential to spawn new drug discovery efforts.

## Organoid-GBM Models

**Figure 1:**
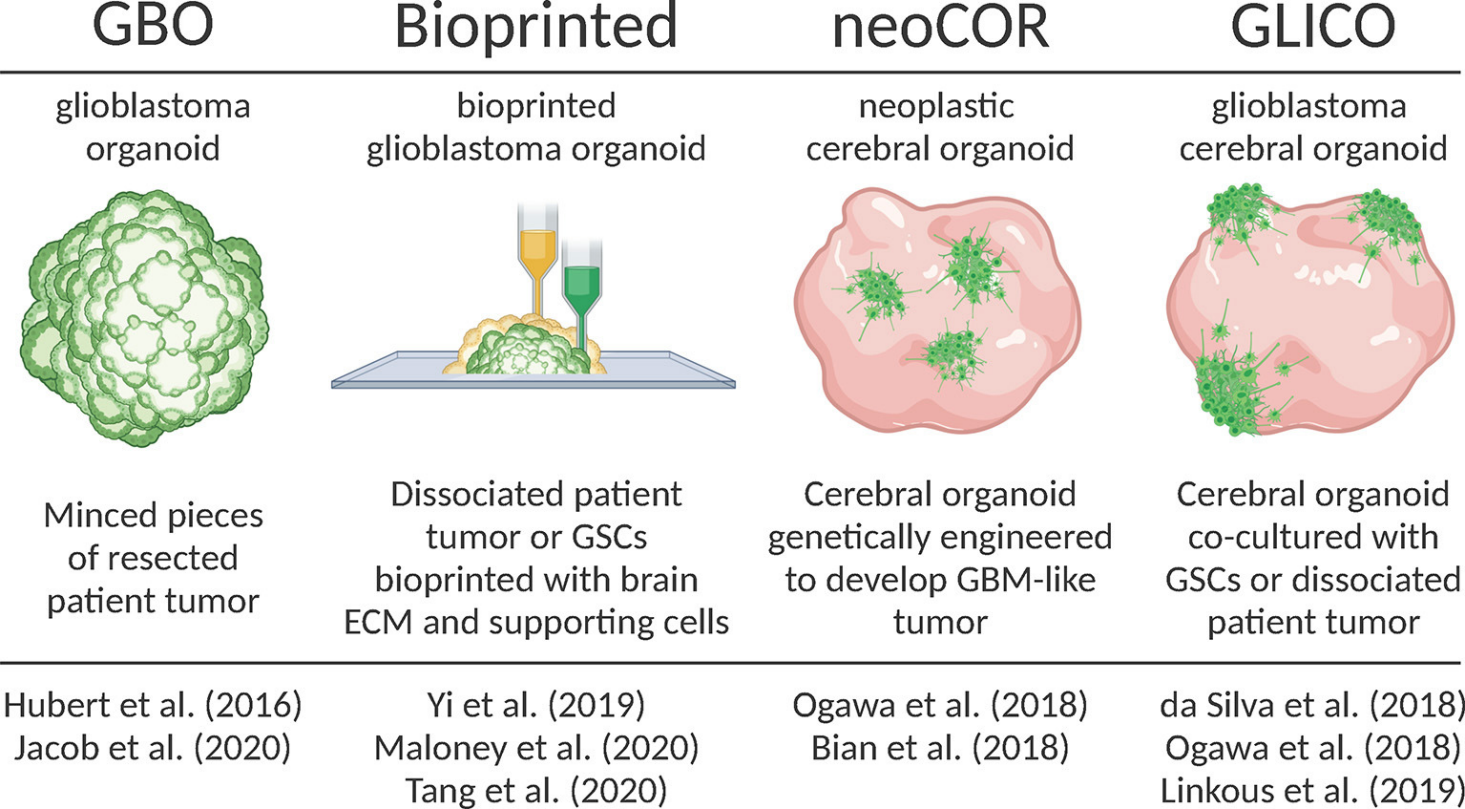
Overview of recently developed organoid-GBM models. Created with Biorender.com.

The last few years have seen an exponential rise in the use of brain organoids as model systems for myriad indications, including GBM. Since the landmark debut of brain organoids in September 2013 (Lancaster et al., 2013) to present time (December 2020) a PubMed search for the term “brain organoids” yields 835 results; of these, 341 (40.1%) were published this year. Clearly brain organoid technology is revolutionizing many areas of neuroscience, particularly in brain development and neurological disease [for review consider Marton and Paşca (2020) and Velasco et al. (2020)]. Here, we review the recent development of different organoid-GBM models and their present and future applications (Figure 1).

### Glioblastoma Organoids [GBO]

Hubert et al. were the first to report culturing GBM as a more complex “organoid” composed of multiple cell types rather than relatively homogenous 3D spheroids (Hubert et al., 2016). They successfully created these GBM organoids from patient-derived primary cultures, xenografts and genetically engineered glioblastomas using matrigel-based 3D culture methods. Additionally, they created GBM organoids directly from patient samples by finely mincing and encapsulating GBM tissues in matrigel for extended periods of time *in vitro*. They observed that GBM organoids recapitulate GBM features including cellular morphology and spatial distribution, hypoxic gradients and resistance to radiation.

Subsequently, Jacob et al. improved upon this model and coined the term GBOs to describe these glioblastoma organoids (Jacob et al., 2020). They radically reduced the time needed to establish GBOs, further characterized their histological features, cellular diversity, transcriptional profiles and developed a protocol for cryopreserving GBOs. In all cases, GBOs remained remarkably similar to the patient samples from which they were derived. Additionally, when orthotopically injected into mice, GBO xenografts displayed extensive invasion into surrounding brain tissues. They also showed that GBOs recapitulate patient-specific responses to treatment by exposing GBOs to similar post-operative treatments as patients (i.e., radiation and TMZ). After treatment they assessed tumor progression in GBOs by calculating the percentage of Ki67+ cells and saw that those GBOs most resistant to treatment corresponded to patients with below median survival. Likewise, GBOs that responded best to treatment corresponded to patients with extended survival. Jacob et al. also showed that chimeric antigen T (CAR-T) cell immunotherapy was most effective for patient samples with a deletion variant in the epidermal growth factor receptor (EFGRvIII). These results support other recent work (O'Rourke et al., 2017; Goff et al., 2019) and provide intriguing support for the use of GBOs in personalized medicine. The aggressiveness of GBM leaves a limited therapeutic window for patient-specific treatments. Thus, the short timeframe needed to establish GBOs and test personalized therapies is a major strength of the model. Weaknesses include a lack of normal brain tissue microenvironment, lack of vasculature and limited residual immune cells.

### Genetically Manipulated Cerebral Organoids [neoCOR]

In 2018, two groups developed protocols for genetically manipulating cerebral organoids to induce GBM tumor growth (Bian et al., 2018; Ogawa et al., 2018). Bian et al. named this model neoplastic cerebral organoids (neoCORs). Both groups used CRISPR-based genome editing techniques to either create oncogenic mutations or induce the expression of oncogenes to cause tumorigenesis within developing iPSC-derived brain organoids. They tracked tumor growth with fluorescent reporters and showed that these tumors recapitulate key features of patient tumors including aggressive invasion when transplanted into the brains of mice. Ogawa et al. (2018) also co-cultured brain organoid-derived tumors with mature organoids and observed invasion that roughly correlated with the degree of lethality in mice.

It is important to note that, while GBOs and neoCORs are both referred to as “organoids,” a key difference between these models is that GBOs are almost entirely tumor and could, therefore, be considered “tumoroids.” On the other hand, neoCORs are tumors formed within cerebral organoids derived from iPSCs that resemble a normal developing human brain. In that sense, the neoCOR model is the organoid equivalent of genetically manipulated *in vivo* models and share the same unique strength: the ability to study the earliest stages of tumorigenesis. Another strength is that, as opposed to GBOs, neoCORs contain both healthy and tumor tissues providing the opportunity to study tumor-brain interactions. Although neoCORs exhibit key GBM features, it remains to be seen how well brain organoid-derived tumors recapitulate the heterogeneity of *in situ* GBM. Additionally, the use of neoCORs is limited in that only defined mutations/oncogenes have been studied to date, although it may be possible to expand the scope of CRISPR-mediated mutations to identify potentially novel mutations that result in tumorigenesis.

### Cerebral Organoid-Glioblastoma Co-cultures [GLICO]

A month after Ogawa et al. (2018) published their brain organoid-derived GBM model, da Silva et al. published a manuscript demonstrating that GSC spheroids could attach and invade immature brain organoids (da Silva et al., 2018). Their work was the first to show that patient-derived cells co-cultured with brain organoids could be used as a GBM model. The following year in the laboratory of Dr. Howard Fine, Linkous et al. improved on this proof-of-concept and coined the term GLICO to describe these cerebral organoid gliomas (Linkous et al., 2019). They showed how different patient GSCs form unique invasive patterns and establish microtubule networks within the internal space of organoids. The formation of similar microtubule networks in the human brain is a characteristic feature of GBM that provides routes of invasion and proliferation, facilitates intercellular communication, and contributes to radiation resistance (Osswald et al., 2015). Using a luciferase-based assay to measure proliferation, Linkous et al. found that different GLICO tumors have differential sensitivities to chemotherapy and radiation.

The GLICO model combines benefits of both the GBO and neoCOR models because GLICOs are created using patient-derived GBM cells and brain organoids, thereby providing the unique capability to investigate tumor-brain interactions (potentially with GBM cekks and organoids derived from the same patient). On the other hand, the GLICO model suffers from similar weaknesses as other organoid-GBM models in that they lack vascularization and immune cells.

### Bioprinted GBM Organoids

Remarkable advancements have been made in the ability to bioprint complex tissues, including 3D models of GBM and other cancers. In 2019, Yi et al. (2019) published a new approach for creating a 3D bioprinted glioblastoma-on-a-chip model from patient-derived cells that contain multiple cell types, different tumor compartments and hypoxic gradients. To accomplish this, Yi and colleagues combined dissociated GBM cells from resected tumors with a decellularized porcine brain “bioink” composed of ECM proteins. After bioprinting GBM cells, they printed a layer of human umbilical vein endothelial cells (HUVEC) with the same porcine bioink. Imaging of GBM cells labeled with a fluorescence dye showed evidence of invasion into surrounding endothelial cells. Immunostaining for pimonidazole (PM), a marker of hypoxia, revealed a hypoxic gradient throughout the bioprinted organoid. These features suggest that bioprinted GBM organoids recapitulate important tumor features.

Recently, other groups made improvements upon the bioprinted GBM model. Maloney et al. published a novel immersion bioprinting method to facilitate drug screening with bioprinted GBM organoids (Maloney et al., 2020). Tang et al. incorporated multiple cells types to form a “tetra-culture” including bioprinted macrophages to model immune interactions (Tang et al., 2020). Overall, the bioprinted model balances the benefits of other organoid models by providing a defined brain extracellular matrix to mimic the brain microenvironment, multiple cell types and defined tumor regions. Perhaps the most important strength of this model is the short timeframe (1–2 weeks) and scalability, making it particularly amenable to drug screening (discussed in more detail below). Future advancements in biomedical engineering are sure to improve on this model. As it is, the lack of normal brain tissue, imprecision of printing, homogeneity of printing substrates and the need for specialized equipment impede the widespread adoption of bioprinted GBM organoids.

## Applications of Organoid-GBM Models

### Importance of Tumor Microenvironment

Using scRNA-seq, Dr. Howard Fine's group compared multiple GBM models: GSC spheroid, GBO, GLICO, and PDX (Pine et al., 2020). In this work Pine et al. performed unbiased hierarchal clustering of transcriptomes from each model and the primary tumor from which they were derived. They showed that the GLICO and PDX derived transcriptomes cluster more closely to primary tumors than GBO or GSC spheroid models. For models containing both GBM and non-GBM cells (i.e., the PDX and GLICO models), GBM cells were isolated by FACS to ensure appropriate comparisons. Pine and colleagues also examined differentially expressed genes across the different GBM models. They found that GLICO tumors had significantly higher expression of key GBM-related genes including SRY-box Transcription Factor 4 (SOX4), Karyopherin Subunit Alpha 2 (KPNA2) and Brevican (BCAN), a marker for GBM invasiveness (Darmanis et al., 2017). Pine et al. also assigned cell types to individual cells based on the cell type signatures for the four cellular states described by Neftel and colleagues (Neftel et al., 2019). They discovered that the distribution of cellular states in the GLICO tumors most closely resembled that of the original patient sample with enrichment of the OPC-like and NPC-like states. Importantly, when GLICO tumors were dissociated and re-plated in 2D they lost many of these important attributes—suggesting that the tumor microenvironment plays a crucial role in maintaining the cellular diversity observed in GBM *in situ*. Overall, the work of Pine and colleagues is an important step in the standardization of different organoid-GBM models.

### Identifying Unique Tumor Cell Types

In addition to classifying GBM cells into established profiles, the GLICO model was recently used in the characterization of a novel GSC subtype. Bhaduri et al. published a GBM tumor cell atlas where primary tumor gene expression profiles were compared to the developing and adult human brain at single-cell resolution (Bhaduri et al., 2020). With this atlas they discovered a new tumor cell type resembling outer radial glia (oRG) cells that have critical roles in the developing human brain. In conjunction with FACS, they used a marker for oRG cells, protein tyrosine phosphatase receptor Z1 (PTPRZ1), to isolate the oRG-like population. They observed that the isolated oRG-like cells undergo mitotic somal translocation—a developmental phenomenon characteristic of their endogenous oRG counterparts in the developing brain (Pollen et al., 2015). This is consistent with other discoveries suggesting that GBM mimics developmental programs (Couturier et al., 2020). Additionally, they showed that *in vitro* knockdown of PTPRZ1 in dissociated primary tumor samples and PDX lines attenuates invasion. To further characterize these oRG-like cells, they co-cultured primary GBM samples sorted into PTPRZ1 positive and negative sub-populations with cerebral organoids. In this experiment, they performed scRNA-seq before and 2 weeks after co-culturing and found that both the PTPRZ1 positive and negative populations gave rise to a more diverse set of cell types than in the original sorted population—supporting the ability of the oRG-like population to reconstitute other cell types within the tumor. One limitation of this work is that while Bhaduri and colleagues observed invasion using the GLICO model they resorted to traditional 2D invasion assays to quantify invasion. Taken together, this work highlights the versatility of organoid-GBM models for use in basic and translational research.

### Invasion

An exciting aspect of emerging organoid-GBM models is the ability to examine GBM invasion in a complex 3D system that approximates the human brain microenvironment. Multiple groups, including our own, have recently developed methods for quantifying GBM invasion in brain organoids. Goranci-Buzhala et al. described a versatile array of methods for modeling GBM invasion with cerebral organoids (Goranci-Buzhala et al., 2020). They demonstrated that iPSCs can be supplemented with fluorescently-labeled GSCs at the beginning of organoid development. They also showed that GSCs can be co-cultured with mature organoids as single cells or 3D aggregates (similar to the GLICO model). After clearing and confocal imaging, they quantified invasion using the number of invasive protrusions and length of microtubules from 2D maximum intensity projection images. In a proof-of-principle experiment, they showed that inhibition of disintegrin and metalloproteinase domain-containing protein 10 (ADAM10) slowed GSC spheroid integration into brain organoids, suggesting these invasion assays could be used to screen for drugs targeting GBM infiltration. Krieger et al. also recently published a method of quantifying invasion in 3D (Krieger et al., 2020). Using ImageJ they calculated the locations of voxels containing fluorescently-labeled GBM cells and their distance from the surface of the organoid. Data from ImageJ was then exported and analyzed with the programming language, MATLAB. They also quantified the number and length of microtubules using image tracing in 3D.

Building upon these findings, we recently characterized a novel BET (Bromodomain and Extra-terminal Domain) inhibitor, UM-002, in cerebral brain organoids (Jermakowicz et al., unpublished findings). We demonstrate that UM-002 is brain-penetrant and reduces GBM cell proliferation *in vitro* and *ex vivo* in an organoid-GBM model similar to the GLICO model described above. We developed an image-based assay to quantify both invasion and proliferation of fluorescently-labeled GBM cells co-cultured with brain organoids. Using Imaris software, we used a semi-automated image analysis pipeline to rendered Z-stack images of organoids in 3D allowing us to compute the distance of each individual GBM cell to the nearest organoid surface. With the ability to differentiate between compound effects on proliferation vs. invasion, we found that UM-002 primarily affects proliferation and not invasion. Further, we utilized the assay for dose-finding by identifying compound concentrations that maximally reduced GBM proliferation without negatively impacting the organoids themselves.

### Personalized Medicine

Recently, Loong et al. published a proof-of-concept for personalized treatment of GBM using GBM organoids (Loong et al., 2020). This work presents a GBM case where the resected tumor was dissected, and a portion of the tumor was used to create GBOs while another part was used for targeted capture sequencing to quantify expression levels of important GBM genes and druggable targets. An RNA sequencing analysis revealed mutation and copy loss of the PTEN (phosphatase and tensin homolog) gene suggesting activation of the mTOR pathway—a common occurrence in GBM. Next, they used patient-specific GBOs to test a panel of FDA-approved treatments for GBM with an emphasis on therapies designed to block mTOR signaling. Similar to the patient, GBOs were resistant to TMZ and everolimus was selected as an alternative treatment. Ultimately, this work provides an important proof-of-concept for individualized treatment of GBM using *ex vivo* systems, however, the prohibitive costs are yet to be addressed. Utilizing a standardized, non-individualized organoid host to test candidate therapies against GBM specimens may offer cost-savings. In so doing, it would be critical to employ integrated multi-omics approaches to characterize individual GBM tumors and organoid-GBM cultures to ensure disease relevance (Stathias et al., 2018; Jermakowicz et al., unpublished findings).

## Future Applications

### Choosing the Right Model

Many different models of GBM have been utilized over the last 50 years, but they have failed to yield life-saving therapies. It is clear that no single model system can sufficiently address the myriad complexities of GBM. Nevertheless, the emergence of organoid-GBM models has begun to fill an important niche between 3D GSC aggregates and *in vivo* models. Importantly, these organoid-GBM models provide a less expensive, higher-throughput method to study GBM without sacrificing a key feature of disease—tumor cell proliferation and dissemination within a human brain microenvironment. Considering the strengths and weaknesses of each method is critical to selecting the most appropriate model for a particular investigation. For example, when studying interactions between the immune system and GBM one might opt for a syngeneic model due to the presence of an intact immune system, albeit murine. Likewise, when studying GBM-microenvironment interactions or GBM invasion, an attractive candidate is likely the GLICO model that combines healthy human cerebral organoids with GBM cells. On the other hand, when considering extensive compound screening, the shorter timeframes and higher throughput of the GBO and bioprinted GBM models are advantageous. Alternatively, the neoCOR model is best-suited for researching specific mutations or neoplasia. Overall, because each GBM model has individual strengths and weaknesses the biological question being asked will dictate the most appropriate model. In addition, as we begin to answer increasingly complex questions, validation using multiple organoid-GBM models is preferable.

### Comparison and Standardization

One consequence of the rapid development of organoid-GBM models is that standardization and reproducibility of various protocols has lagged behind. For example, in generating the GLICO model, Linkous et al. (2019) observed that age of the cerebral organoid did not significantly affect the growth rate of GBM tumors. By contrast, when illustrating different GBM invasion assays with organoids, Goranci-Buzhala et al. (2020) observed that the time for GSCs to integrate into organoids decreased with age, suggesting higher rates of invasion in more mature organoids. There are multiple rationalizations that could explain this discrepancy. For instance, they could arise from differential proliferation or invasion rates, or other unknown differences between GSC lines. Whatever the case, our collective efforts to improve upon the various systems should go forward with transparency and recognition of the need to maximize standardization of protocols, particularly since various labs will utilize unique primary GBM cells and iPSC lines.

Another example demonstrating the need for comparison and standardization of organoid-GBM protocols is the varied methods used to quantify GBM invasion that have been developed by different research groups. Some have used the number of GBM protrusions or microtubules that are assumed to correlate with invasiveness (Osswald et al., 2015). However, tracing protrusions (especially in 3D) is time-consuming and low-throughput. When images are collapsed into 2D maximum projection views to facilitate image analysis, the spatial organization of GBM cells is lost. Alternatively, we and others have used the location of cells to compute their distance to the surface within organoids as a measure of invasion. While this method is potentially higher throughput, it does not provide information about GBM cell morphology. In the end, some combination of these strategies is likely to be adopted as standard practice. While these two examples may only represent minor discrepancies, they illustrate a potential stumbling block in organoid-GBM modeling that should be addressed early and often by comparison and standardization efforts across research teams.

### Cellular Plasticity and Stemness

GBM recurrence is driven by residual GSCs that escape resection by extensive invasion into surrounding brain tissues. These cells resist subsequent chemotherapy and radiation, at least in part, through remarkable genetic and phenotypic plasticity (Liau et al., 2017; Minata et al., 2019; Neftel et al., 2019). Thus, understanding the mechanisms that fuel GSC plasticity is a major goal of current GBM research. As mentioned above, some groups have begun to use organoid-GBM models to examine GBM subtypes and inter-subtype plasticity. For example, Pine and colleagues showed that the GLICO model best recapitulates the diversity of subtypes seen in patient samples (Pine et al., 2020). Bhaduri et al. (2020) sorted patient samples into separate populations and showed that sorted populations reconstitute much of the heterogeneity seen in the original sample. These studies provide a starting point for future efforts to use organoid-GBM models to study GSC treatment resistance, diversity and plasticity. The analysis of key markers of GSC stemness such as Sox2, CD44, cMyc, and Nestin have yet to be compared among organoid-GBM models, which could provide further insight into which model is best when targeting GSC stemness. In addition, other important and related questions still remain. For example, how well do organoid-GBM models mimic patterns of GBM recurrence after treatment and/or surgery? How does the organoid microenvironment affect GSC plasticity in response to different treatments? Addressing such questions regarding GSC plasticity and stemness are vital next steps in validating organoid-GBM models.

### Incorporating Advancements in Organoid Technology

Organoid technology is improving rapidly with the effective incorporation of immune cells and vascularization representing major hurdles in refining the technology [for review see Marton and Paşca (2020)]. These and other advancements are certain to facilitate new discovery research and drug development efforts (Tang et al., 2020). For example, GBM is known to invade along the perivascular space [Scherer, 1938; see also review by Cuddapah et al. (2014)], therefore, vascularized organoid-GBM co-cultures would be an intriguing model to study invasive behaviors and potential therapies to prevent perivascular invasion. Additionally, multiple brain region-specific organoid protocols have now been developed. It would be interesting to determine how the behavior of GBM differs within different brain region-specific microenvironments. Another limitation of current brain organoid differentiation protocols is that they produce relatively immature neurons and glia and a high percentage of replicating progenitor cells, as compared to developed human brains. Currently, even the most mature and longest-cultured brain organoids resemble fetal brain tissue. This limitation is not unique to brain organoids and efforts have been made to artificially age iPSC-based cultures genetically or pharmacological [Miller et al., 2013; Garcia et al., 2015; for review of how brain organoids are being used to study other age-related disease see Grenier et al. (2020)]. Overall, the future is sure to bring these and other unforeseen advancements in brain organoid technology—the incorporation of which will continue to refine the organoid-GBM model system.

### Compound Screening

**Figure 2:**
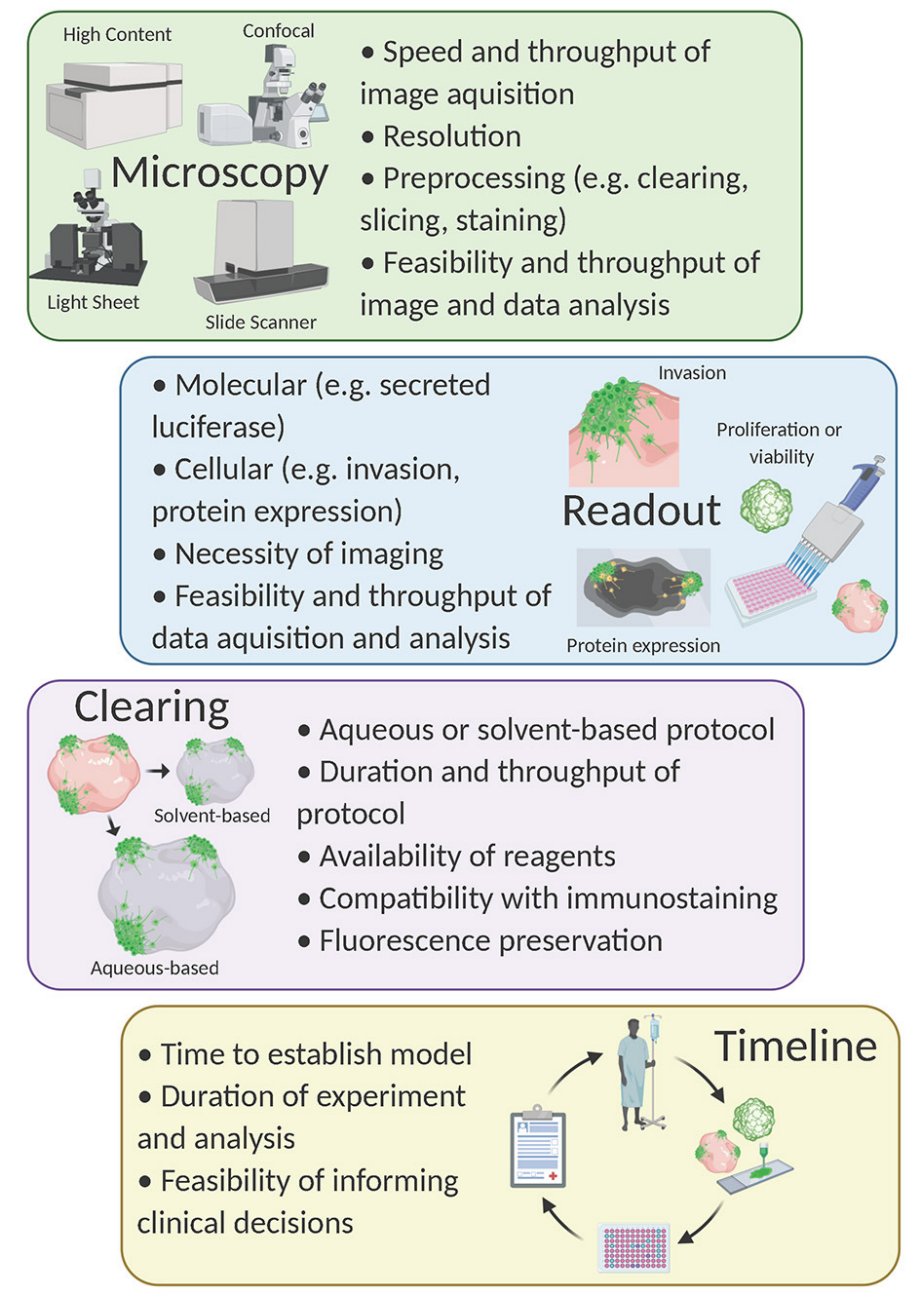
Considerations for compound screening with organoid-GBM models. Created with Biorender.com.

Compound screening with 3D models is an emerging field, but to date, tumor organoids are primarily used for compound screening [for review see Weeber et al. (2017) and Aboulkheyr Es et al. (2018)]. Although some have used patient-derived GBM organoids to validate hits from higher-throughput *in vitro* compound screens (Yi et al., 2018; John Liu et al., 2020), to our knowledge, a large-scale primary drug screen using an organoid-GBM model has not yet been achieved. Nevertheless, multiple groups have performed proof-of-concept experiments demonstrating the feasibility of this strategy in drug discovery (Bian et al., 2018; Linkous et al., 2019; Goranci-Buzhala et al., 2020; Jacob et al., 2020; Loong et al., 2020). These efforts have illuminated some key obstacles and questions that must be addressed moving forward (Figure 2).

Yi et al. (2019) demonstrated that bioprinted GBM organoids can be used to determine the susceptibility of individual patient-derived GBM organoids to different combination therapies. They defined transcriptional profiles of two different primary GBM samples using microarray and found GBM samples with abnormalities in the DNA damage response making them selectively vulnerable to inhibition of the DNA damage response. They subsequently showed that treatment of bioprinted GBM organoids with DNA repair inhibitors (O^6^-benzylguanine, cisplatin, KU60019) and radiation was significantly more effective in cells with DNA damage response abnormalities. Cell viability after treatment was assessed using the colorimetric Cell Counter Kit-8 (CCK-8) assay in which a formazan dye is produced in the presence of electron carriers in living cells.

Another group performed similar experiments using bioprinted GBM organoids (Maloney et al., 2020). Seven days after bioprinting, GBM organoids were treated with multiple concentrations of dacomitinib and NSC59984 (an EGFR inhibitor and small molecule p53 activator, respectively). After 72 h, cell viability was assessed by quantifying ATP levels. They observed that both GBM samples were sensitive to drug treatment but had unique drug concentration-dependent responses. Notably, the CCK-8 assay provided a more reliable assessment of cell viability as compared to the ATP quantification method. While these were small-scale proof-of-concept experiments, they raise important questions about how best to quantify cell viability in complex organoid-based systems—a critical readout in most screening assays.

Using the neoCOR model, Bian et al. (2018) demonstrated that neoCORs are suitable for drug testing. They treated multiple neoCORs with afatinib (an EGFR inhibitor in clinical trials; see NCT02423525). After 40 days of treatment, neoCORs were dissociated and GFP-expressing tumor cells were sorted with FACS. They found that the number of GBM cells was significantly less in neoCORs with EGFR overactivation where the effect of afatinib was robust. While using FACS provides a versatile read-out option, up-scaling for high-throughput screening with complex organoid systems may not be feasible.

Linkous et al. (2019) showed that patient-derived GBM cells cultured with the GLICO model exhibit differential responses to compound treatment. They co-cultured patient-derived GBM cells engineered to secrete luciferase with cerebral organoids for 5 days. After establishment, they treated organoids with two chemotherapeutic agents used for GBM—TMZ and bis-chloroethylnitrosourea (BCNU). Then, at zero- and seven-days post-co-culture, media was collected and analyzed for luciferase levels to approximate the number of viable GBM cells within organoids. They observed that one of the samples responded well to BCNU while the other was resistant and responded better to TMZ. Intriguingly, when the same patient samples were cultured in 2D as GSCs, the two lines were equally susceptible to treatment with TMZ and BCNU. The use of secreted luciferase as a proxy for determining GBM cell counts within organoids offers simplicity, sensitivity and scalability making it amenable to high-throughput drug screening campaigns. The major limitation of the luciferase system (as well as the other cell viability assays mentioned above) is the inability to address more complex features of GBM (e.g., invasion and morphology).

Goranci-Buzhala et al. (2020) co-cultured GSC spheroids with cerebral organoids for 24 h then treated with recombinant human Neuroglin3 (NLGN3) or an ADAM10 inhibitor (GI254023X). NLGN3 contributes to GBM progression—an effect mediated by the sheddase ADAM10 (Venkatesh et al., 2017). They observed that addition of NLGN3 reduced the time needed for GSCs to integrate into the cerebral organoid and that ADAM10 inhibition blocked this effect, significantly extending time to integration. Integration was monitored by time lapse imaging of organoid-GSC co-cultures. This preliminary experiment provides a proof-of-concept for using more sophisticated imaging techniques for compound testing, but time-lapse imaging is not a particularly high-throughput approach.

We and others developed protocols that rely on fluorescence imaging of optically cleared brain organoids (Goranci-Buzhala et al., 2020; Krieger et al., 2020). The basis of all tissue clearing is the equilibration of the refractive index to allow for imaging at greater depths than would otherwise be possible. Dozens of clearing protocols exist and can be divided into aqueous-based protocols (e.g., Scale, FRUIT, PACT) (Hama et al., 2011, 2015; Hou et al., 2015; Neckel et al., 2016; Yu et al., 2017) or organic solvent-based protocols (e.g., FluoClearBABB, 3DISCO, CUBIC) (Ertürk et al., 2012; Schwarz et al., 2015; Susaki et al., 2015). In general, water-based clearing causes tissues to physically expand while solvent-based clearing causes tissues to shrink (Wan et al., 2018). Thus, the choice of clearing method is an important consideration when designing this type of experiment. For example, if examining cellular morphology, tissue expansion might be beneficial. In contrast, one might consider a solvent-based protocol to allow for imaging at greater relative tissue depth (Pan et al., 2016).

Other important considerations when choosing a clearing method include time, materials, compatibility with immunological staining and fluorescence preservation. (1) The time needed to clear organoids can vary depending on the size of the tissue or organoid, but also varies dramatically between protocols (usually water-based protocols are gentler and take longer). (2) Some clearing protocols use materials commonly found in research laboratories; others require specialized chemicals. (3) Solvent-based protocols are generally less compatible with immunostaining due to the dehydration of the tissue. (4) Typically, solvent-based clearing protocols are more likely to result in a quenching of fluorescent proteins while water-based protocols are more likely to retain endogenous fluorescence (Wan et al., 2018). Updated protocols, such as FDISCO, have begun to address this (Qi et al., 2019), however, the loss of fluorescence remains problematic during storage. Since complex organoid-GBM systems present challenges for image-based assays, the choice of clearing protocol becomes an important experimental design consideration.

The choice of imaging platform is also crucial for the development of an organoid-GBM screening assay. Organoid-GBM models with fluorescent reporters can be imaged with epifluorescence, confocal, or light-sheet microscopy (Hubert et al., 2016; Linkous et al., 2019; Goranci-Buzhala et al., 2020; Krieger et al., 2020). Epifluorescence is simple and relatively inexpensive yet lacks the resolution necessary for imaging 3D tissues. Light sheet microscopy is designed for larger tissues and can be adapted for use with organoids, but cannot be scaled for use in high-throughput (Dekkers et al., 2019). Alternatively, confocal microscopy offers sufficient resolution to resolve important read-outs such as location in 3D space, cellular morphology and expression of key marker proteins, but is relatively low throughput. Nevertheless, advancements in software and microscopy are trending toward automated high-throughput 3D confocal-based phenotypic screening (e.g., the Opera Phenix High Content Screening System by PerkinElmer). Combining recent progress in organoid-GBM modeling with advanced high-throughput imaging platforms will be a powerful tool for drug discovery in the very near future. One of the main reasons 3D cellular models have not been used for compound screening is their increased complexity and variability. Since high-throughput compound screening requires robust assays, it is essential to have 3D models that can be consistently produced at scale. Despite these barriers, the lack of success in developing effective drugs using current technologies strongly suggests that researchers will need to embrace more complex models.

## Conclusion

While many important discoveries have been made over the last several decades, GBM remains a lethal cancer with no treatment capable of preventing disease recurrence. Recent research has illuminated GBM as an extremely complex and heterogeneous disease, placing new emphasis on developing more disease relevant model systems. Here, we have summarized the current repertoire of new organoid-GBM models and discussed important goals for their implementation into drug discovery pipelines. While critical improvements are still needed, their ability to more accurately model the complexities of GBM at lower cost and higher throughput suggests they have an auspicious role to play. Implementing this rapidly advancing technology in combination with low-passage patient-specific GBM cells offers new hope that life-saving therapies can be developed.

## Author Contributions

ZZ and MR planned and outlined the scope of work. MR wrote the first draft and produced figures. ZZ made substantial edits to produce a mature manuscript. NA and MI provided feedback and made edits to finalize the document. All authors contributed to the article and approved the submitted version.

## Conflict of Interest

The authors declare that the research was conducted in the absence of any commercial or financial relationships that could be construed as a potential conflict of interest.
